# Gestational diabetes mellitus and maternal-infant microbiome axis: mechanistic insights and therapeutic interventions

**DOI:** 10.1186/s12967-026-08491-6

**Published:** 2026-06-22

**Authors:** Jinjoo Choi, Jihyun Keum, Min‑Jin Kwak, Seung Hyun Kim, Jeong Kyu Hoh, Byong-Hun Jeon, Hyun-Kyung Park

**Affiliations:** 1 Department of Pediatrics, Hanyang University College of Medicine, Seoul, 04763 Republic of Korea; 2 Department of Obstetrics and Gynecology, Hanyang University College of Medicine, Seoul, 04763 Republic of Korea; 3 Department of Forest Products and Biotechnology, Kookmin University, Seoul, 02707 Republic of Korea; 4 Department of Pediatrics, Samsung Medical Center, Sungkyunkwan University School of Medicine, Seoul, 06351 Republic of Korea; 5 Department of Earth Resources and Environmental Engineering, Hanyang University, Seoul, 04763 Republic of Korea

**Keywords:** Dysbiosis, Gestational diabetes mellitus, Gut, Microbiome, Neonate

## Abstract

**Background:**

Gestational diabetes mellitus (GDM) alters maternal metabolism and the gut microbiota, thereby significantly affecting neonatal health. This narrative review synthesizes current clinical and translational evidence on temporal changes in the maternal gut microbiota across the three pregnancy trimesters in women with GDM and examines its subsequent impact on neonatal microbiome composition, immune development, and long-term disease risk.

**Main body:**

GDM-associated dysbiosis has been reported early in pregnancy, but human evidence remains heterogeneous and does not establish direct vertical transmission; instead, maternal metabolic status and perinatal exposures may jointly shape early neonatal microbial patterns and immune–metabolic trajectories. These microbial alterations are closely associated with immune dysregulation and increased risks of inflammatory and metabolic disorders in offspring. Although diet, obesity, and probiotics influence microbial composition, their clinical efficacy in GDM remains inconsistent. Importantly, the available evidence remains heterogeneous, reflecting differences in cohort characteristics, sequencing methods, and study design, which limits definitive causal interpretation.

**Conclusions:**

Advances in microbiota-based diagnostics and personalized microbial interventions—including microbiome-guided dietary modulation, targeted probiotic strategies, and metabolite-focused approaches tailored to individual maternal metabolic and microbial profiles—offer promising strategies to mitigate adverse outcomes by targeting the maternal–neonatal microbiome axis, highlighting microbiome-based approaches as an emerging translational direction.

**Clinical trial number:**

Not applicable.

**Graphical Abstract:**

**Figure:**
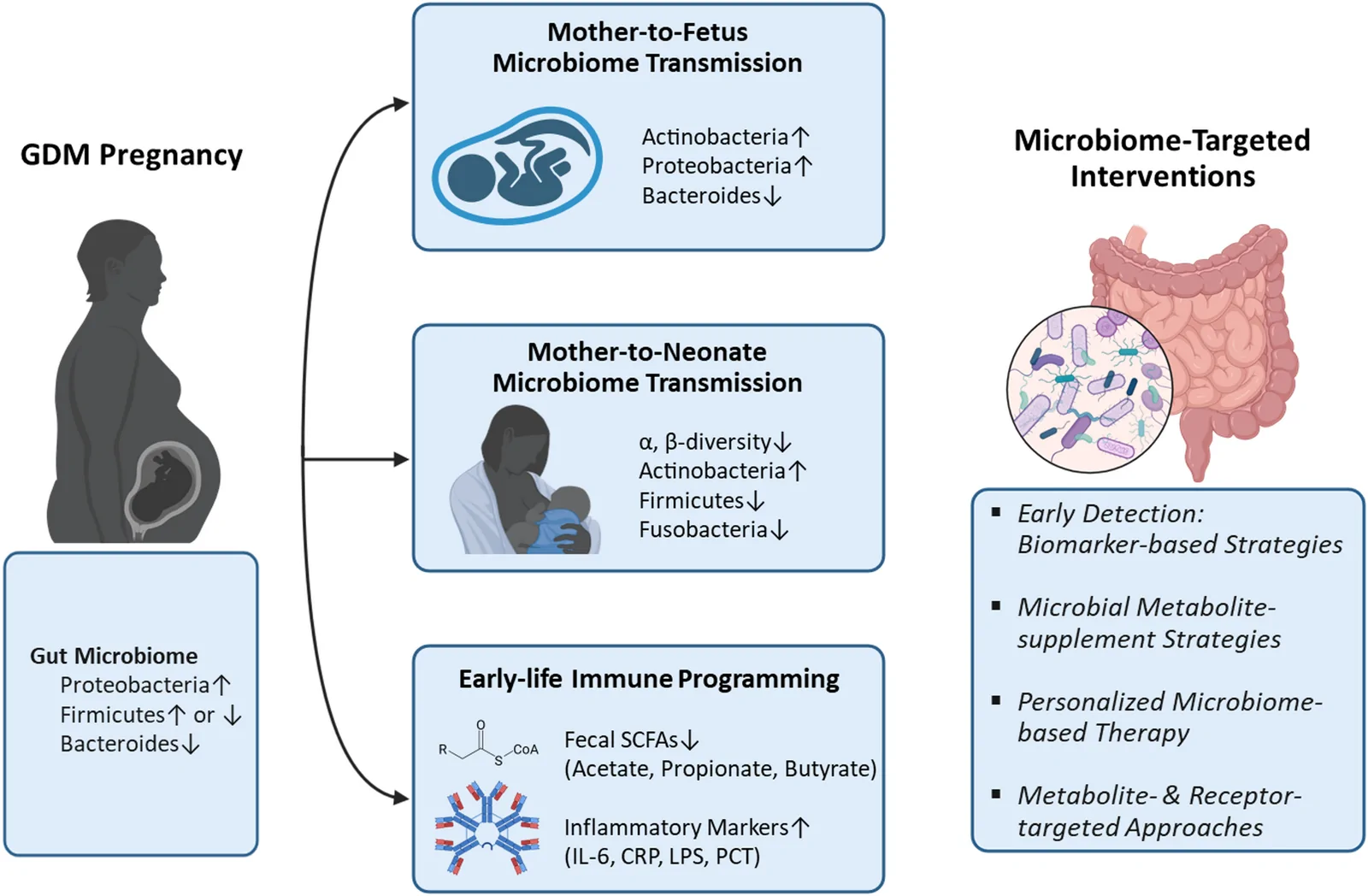

## Gestational diabetes mellitus as a maternal-neonatal microbiome axis

Gestational diabetes mellitus (GDM) is recognized as a multifaceted metabolic disorder. It extends beyond simple glucose dysregulation to involve complex interactions among maternal immune, endocrine, and microbial systems. Affecting nearly one in seven pregnancies worldwide, GDM is more than a transient metabolic disturbance. It is a key determinant with long-term implications for the mother’s and offspring’s health [1].

Pregnancy itself is characterized by a progressive insulin resistance induced by placental hormones, including human placental lactogen, progesterone, and cortisol. Under normal glucose tolerance, pancreatic β-cell hypersecretion compensates for this resistance to maintain euglycemia [2]. However, in GDM, insulin resistance causes β-cell dysfunction and leads to persistent hyperglycemia and overloading of the β-cells. Consequently, metabolic disorders associated with glucotoxicity are accompanied by significantly increased adipocyte fatty acid-binding protein (FABP) expression during pregnancy, decreased peroxisome proliferator-activated receptor gamma (PPARγ) expression, insulin receptor substrate (IRS-1) dysfunction, and chronic inflammation mediated by insulin receptor phosphorylation [3]. These metabolic and inflammatory perturbations disrupt gut barrier integrity and immune tolerance. This process reshapes the maternal gut microbiome and alters the intrauterine environment [4].

The maternal-neonatal microbiome axis consists of interconnected biological compartments. These include the maternal gut microbiome, circulating metabolites, the placental interface, amniotic environment, and the developing fetal and neonatal microbiome. These compartments are functionally linked by endocrine signaling, immune mediators, and microbiota-derived metabolites such as short-chain fatty acids (SCFA) and bile acids. Emerging evidence suggests that maternal microbial dysbiosis plays a critical role in fetal development. This dysbiosis is characterized by the depletion of s SCFA-producing bacteria and the enrichment of pro-inflammatory taxa [5]. These shifts may influence fetal via metabolite- and inflammation-mediated pathways. Proposed mechanisms include transplacental exposure to microbial-derived metabolites, cytokines, and bacterial components. Additionally, perinatal microbial seeding occurs during delivery and early postnatal feeding [6–8]. Such transmission shapes early microbial colonization, immune maturation, and metabolic programming. Ultimately, it may predispose offspring to inflammatory and metabolic diseases later in life [9]. Understanding GDM through the maternal–neonatal microbiome axis provides new insights into disease pathogenesis and intergenerational risks [4]. This perspective also identifies targets for microbiota-based interventions. These include probiotics, prebiotics, supplementation with metabolites, and personalized nutrition. The goal of these strategies is to restore microbial balance and reduce the long-term consequences of GDM.

To provide conceptual clarity, this review adopts a structured narrative framework. We first examine GDM- induced maternal microbiome shifts and their heterogeneity across trimesters. Subsequently, we evaluate maternal-fetal microbial interactions and their role in early-life immune programming. By synthesizing human and mechanistic studies, we discuss long-term outcomes for offspring. Finally, we assess emerging therapeutic strategies, such as probiotics and precision nutrition. This review aims to bridge mechanistic insights with clinical relevance to mitigate the intergenerational impact of GDM.

## GDM-induced maternal microbiome shift

Pregnancy is an enthralling biological process that entails several physiological alterations, many of which are well recognized, including hormonal and metabolic changes. However, the significance of the microbiota in gestation has only been recognized in the last decade. Microbiome communities residing in the body are considered among the most essential contributors to host metabolic and immunological health.

### Gut microbiome alterations in GDM compared with healthy pregnancy

During a healthy pregnancy, the maternal gut microbiome might remain relatively stable or exhibit an increased abundance of Proteobacteria and Actinomycetota, a reduction in butyrate-producing bacteria, and lower microbial richness in late gestation [10, 11]. Conversely, findings on GDM-specific alterations remain inconsistent. The heterogeneity across studies reflects multiple methodological and clinical sources of variation. These include differences in diagnostic criteria for GDM, gestational age at sampling, sequencing platforms, and statistical adjustment for key confounders such as maternal ethnicity, baseline BMI, dietary patterns, antibiotic exposure, and glycemic control. Some studies reported minimal differences in gut microbial profiles between GDM and non-GDM pregnancies [11]. Whereas others have described compositional shifts within Firmicutes, Bacteroides, and Actinomycetota. Metagenomic analyses further suggested greater variability in metagenomic linkage groups (MLGs) in GDM. Functional enrichment analyses indicate that GDM-associated microbiomes are characterized by increased membrane transport, energy metabolism, and lipopolysaccharide-related pathways, whereas Genera such as *Parabacteroides*, *Eggerthella*, *Megamonas*, and *Allofustis* were positively correlated with glycemic control, whereas *Alistipes* was negatively correlated with glucose tolerance. Functional analyses indicated that GDM-associated microbiomes are enriched in membrane transport, energy metabolism, and lipopolysaccharide pathways, whereas amino acid metabolism was more prominent in non-GDM controls [12].

In addition to gut microbial dysbiosis, literature indicates that microbiome changes also occur in the oral cavity, vagina, pharynx, and amniotic fluid of GDM mothers [13, 14]. Furthermore, the placental microbiota has also been reported to differ structurally and functionally in GDM. Placentas from women with GDM exhibited increased abundance of Firmicutes and decreased abundance of Proteobacteria and Bacteroides compared with those from non-GDM women. A reduced placental abundance of *Acinetobacter* was associated with lower eosinophil counts in the latter two trimesters of pregnancy, decreased placental synthesis of IL-10 and TIMP3, and higher maternal glucose levels in the O’Sullivan test [15]. Moreover, Firmicutes and Proteobacteria in the gut and placenta have been associated with liver cancer in some literature, so attention should be paid to the related prognosis in children in the future [16]. However, the interpretation of a placental microbiota requires a critical discussion regarding potential sample contamination and the inherent methodological limitations of low-biomass sequencing [17].

### Trimester-specific gut microbial dysbiosis in women with GDM

Several studies have reported gestational age–dependent changes in the maternal gut and vaginal microbiomes throughout pregnancy [18, 19]. Early pregnancy is characterized by immune tolerance and metabolic adaptation supporting placentation. Shifts in the gut microbiome during early pregnancy have been proposed as potential biomarkers for GDM development. Although many of these findings derive from exploratory and relatively small-scale studies—limiting firm conclusions regarding diagnostic sensitivity or specificity—recent longitudinal investigations have observed elevated inflammatory cytokine levels, reduced fecal short-chain fatty acids (SCFAs), and early microbial compositional changes in women who later develop GDM. Notably, some of these alterations have been detected up to 10 weeks prior to clinical diagnosis, suggesting that metabolic–microbial perturbations may precede overt hyperglycemia [6]. Collectively, these observations highlight the potential of early-pregnancy microbiome and metabolite profiling as predictive tools; however, large, well-designed prospective cohorts are required to validate their clinical utility.

**Fig. 1:**
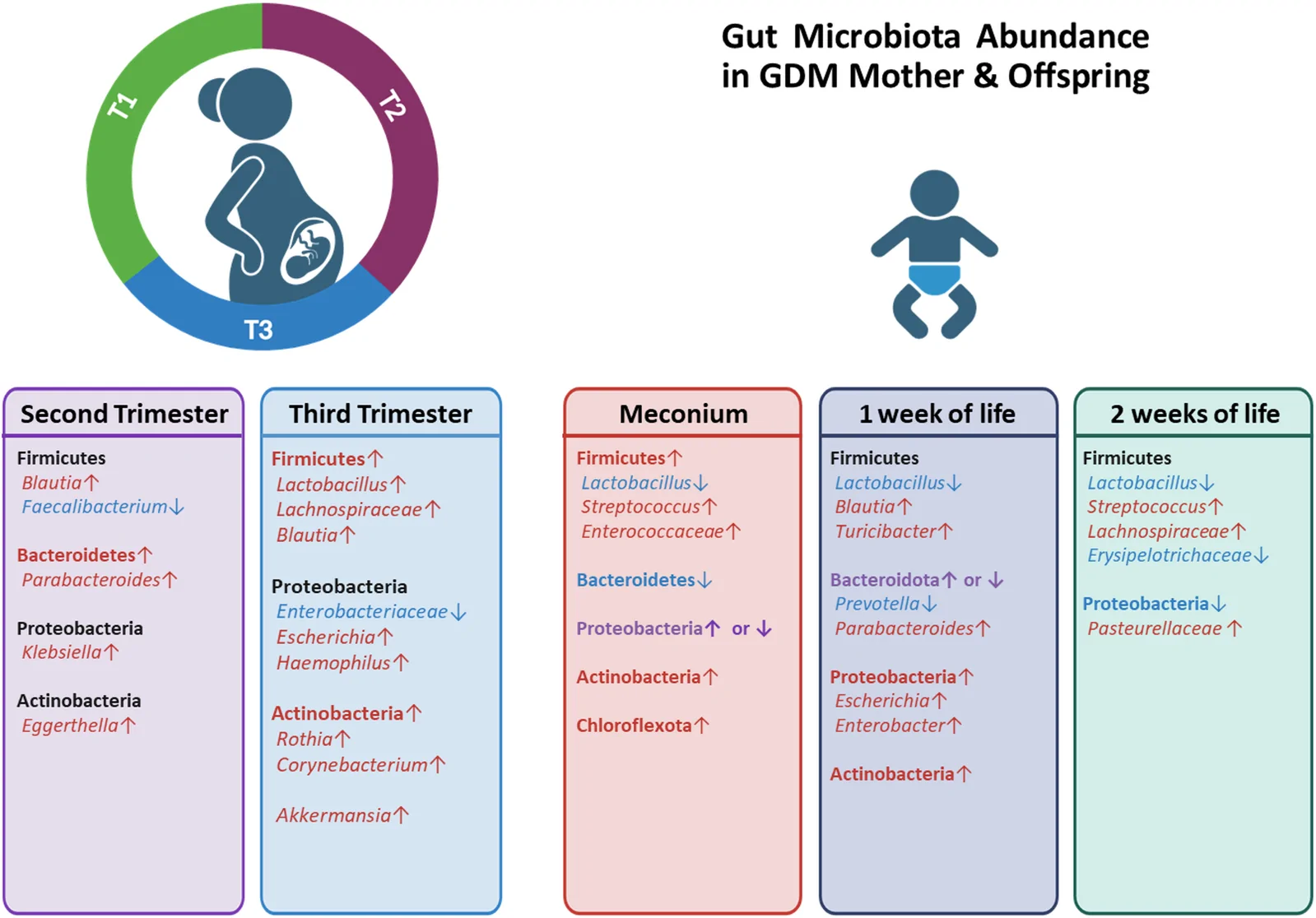
Gestational diabetes mellitus (GDM) reshapes maternal and neonatal gut microbiota during pregnancy (second and third trimesters) and early-life (meconium, first and second weeks of life). Gut microbial composition is changed by gestational diabetes mellitus throughout pregnancy (2nd and 3rd trimester), and neonatal development (meconium, 1st week and 2nd week of life). During mid-pregnancy, *Blautia* [90], *Parabacteroides* [12], *Klebsiella* [12], and *Eggerthella* [94] increase while *Faecalibacterium* [90] decreases. In late pregnancy, *Lactobacillus* [89], *Lachnospiraceae* [90], and *Blautia* [90] increase, with the increment of *Escherichia* [89], *Haemophilus* [93], *Rothia* [41], *Corynebacterium* [91], and *Akkermansia* [22]. In neonatal meconium, the population of *Lactobacillus* [39] and *Bacteroidetes* [37] decreases; however, *Streptococcus* [5] and *Enterococcaceae* [5, 13] increase due to maternal GDM. In the first week of a newborn infant’s intestine, *Lactobacillus* [40] and *Prevotella* [31, 40, 41] decrease, while *Blautia* [40], *Turicibacter* [41], *Parabacteroides* [40], *Escherichia* [40], and *Enterobacter* increase [13]. Maternal GDM reduces the populations of *Lactobacillus* [51] and *Erysipelotrichaceae* [51], with an increase in the populations of *Streptococcus* [51], *Lachnospiraceae* [51], and *Pasteurellaceae* [51]. The microbial shifts summarized in this figure are derived from studies with heterogeneous designs, sample sizes, and sequencing or analytical platforms, as discussed in the main text. Proteobacteria in meconium and Bacteroidetes in the first week of life showed inconsistencies across studies

In the second trimester, when physiological insulin resistance peaks [20], microbial alterations may interact more directly with host metabolic pathways. Increased abundance of *Holdemania*, *Megasphaera*, and *Eggerthella* and reduced *Flavonifractor*, *Streptococcus*, and *Coprococcus* have been observed in GDM [12, 13]. Specifically, the abundance of *Streptococcus* and *Coprococcus* was consistently reduced in women with GDM in both the first and second trimesters (Fig. 1; Table 1) [21]. Women with GDM also exhibited a higher abundance of Bacteroidetes at the phylum level alongside a lower abundance of *Corynebacteriales* at the order level and *Nocardiaceae* and *Desulfovibrionaceae* at the family level. Genus-level comparisons revealed a higher prevalence of *Bacteroides*, *Weissella*, *Fusicatenibacter*, *Parabacteroides*, *Roseburia*, and *Flavonifractor* (Fig. 1; Table 1) [22]. Changes in SCFA-producing genera such as *Faecalibacterium* and *Prevotella*, are associated with overexpression of pro-inflammatory species, and these microbial community alterations correlate with serum metabolic markers. Notably, dopamine deficiency and elevated 2-hydroxybutyric acid levels are prominent, consistent with glucose metabolism parameters [23].

**Table 1:** Comparison of the microbiome of women with and without GDM and their offspring^1^

Gut Microbiome			GDM									
			Mother						Neonate			
Phylum	Family		Genus		T2, T3		T3		Meconium		1st week	2nd weeks
Firmicutes	Lactobacillaceae		*Lactobacillus*				↑[89]		↓[39]		↓[40]	↓[51]
	Streptococcaceae		*Streptococcus*						↑[5]			↑↑[51]
	Enterococcaceae								↑[5, 13]			
	Carnobacteriaceae		*Isobaculum*				↑[88]				↑[41]	
	Lachnospiraceae		*Blautia*		↑[90]		↑[13, 91]				↑[40]	↑[51]
	Oscillospiraceae		*Faecalibacterium*		↓[90]		↑[24],↓[13]					
	Erysipelotrichaceae											↓[51]
	Turicibacteraceae		*Turicibacter*								↑[41]	
	Coprobacillaceae		*Catenibacterium*		↑[12]							
Bacteroidetes					↑[91, 92]				↓[37]		↑[40],↓[41]	
	Prevotellaceae		*Prevotella*								↓[31, 40, 41]	
	Tannerellaceae		*Parabacteroides*		↑[12]						↑[40]	
Proteobacteria							↑[93]		↑[37, 39],↓ [5]		↑[5]	↓[51]
	Enterobacteriaceae						↓[13]				↓[31]	
			*Escherichia*				↑[89]				↑[40]	
			*Enterobacter*								↑[13]	
			*Klebsiella*		↑[12]							
	Pasteurellaceae											↑[51]
			*Haemophilus*				↑[93]					
Actinobacteria									↑[13]		↑[28, 40]	
	Eggerthellaceae		*Eggerthella*		↑[94]							
	Coriobacteriaceae		*Collinsella*				↑[41]					
	Micrococcaceae		*Rothia*				↑[41]					
	Corynebacteriaceae		*Corynebacterium*				↑[91]					
Fusobacteriota											↑[28]	
Chloroflexota									↑[39]			
Verrucomicrobiota	Akkermansiaceae		*Akkermansia*				↑[22]					

T, trimester; 1st week, The first week of life

^1^reported microbial changes reflect aggregated findings from heterogeneous study populations and methodologies and should be interpreted in light of the sources of variability discussed in the main text

During the third trimester, both the gut microbiomes of women with GDM featured a higher abundance of Firmicutes and a lower abundance of Bacteroidetes and Actinomycetota compared with the findings in women without GDM (Fig. 1; Table 1) [24]. In GDM, the abundant *Bacteroides vulgatus* and *Ruminococcus gravus* from the early stages of pregnancy showed a significant positive correlation with insulin signaling pathways and lipopolysaccharide biosynthesis increasingly toward the third trimester. These findings suggest a potential association between the gut microbiota and the development of GDM [25].

### Sources of heterogeneity across GDM microbiome studies

A comprehensive review of the studies summarized in Tables and Figures indicates that research findings on changes in microbial composition, diversity, and phylum-level shifts in GDM remain heterogeneous. Several methodological and clinical factors may contribute to this inconsistency. These include differences in study design (cross-sectional versus longitudinal sampling), population characteristics (ethnicity, baseline BMI, dietary patterns), GDM diagnostic criteria, gestational age at sampling, sequencing platforms, and the extent of adjustment for key confounders such as antibiotic exposure, probiotic use, delivery mode, and glycemic control.

Importantly, analyses involving low-biomass specimens—such as placental, intrauterine, and neonatal samples—are particularly susceptible to contamination, batch effects, and reagent-derived microbial signals. These technical challenges may contribute to discrepancies across datasets and underscore the need for standardized sampling protocols, rigorous negative controls, and harmonized analytic frameworks in future GDM microbiome research.

## Impact of GDM on early-life microbiota development

The maternal gut microbiome is influenced by GDM, and maternal hyperglycemia has been associated with alterations in maternal and early neonatal microbial profiles; however, current evidence suggests that these changes likely reflect a combination of prenatal factors and shared postnatal environmental exposures rather than direct maternal transmission alone [6, 8, 26].

Postnatal influences, including delivery mode, breastfeeding, antibiotic exposure, and the shared maternal–infant environment, have been shown to substantially shape early microbial colonization and may partially explain neonatal dysbiosis observed in infants born to mothers with GDM [27, 28].

Moreover, although experimental and translational studies continue to explore potential intrauterine microbial or metabolite-mediated effects, recent reviews emphasize that human evidence remains limited and susceptible to contamination bias in low-biomass samples, necessitating cautious interpretation of in utero colonization hypotheses [29, 30].

Importantly, most human studies remain cross-sectional or short-term, and the limited availability of longitudinal follow-up data restricts definitive conclusions regarding the persistence of neonatal dysbiosis and its long-term clinical consequences [31, 32]. Current evidence supports an association between maternal GDM and early-life microbial alterations, but causality, persistence, and long-term impact remain areas requiring carefully designed longitudinal cohort studies integrating environmental and host-related factors [6, 33].

### Mother-to-fetus microbiome transmission in GDM

#### In utero microbial transmission from mothers with GDM

Intrauterine microbial transmission has been proposed as a potential mechanism linking maternal dysbiosis to fetal immune and metabolic programming. Several studies have reported the detection of bacterial DNA in placental tissue, amniotic fluid, or meconium, including taxa belonging to *Firmicutes*, *Tenericutes*, *Proteobacteria*, *Bacteroidetes*, and *Fusobacteria* [34]. In the past, it was believed that the uterus was sterile, but recent studies have shown that microbiota exist in the placenta and amniotic fluid, particularly Proteobacteria. Interestingly, a report exists indicating that the microbiome of the baby’s meconium, the first stool after birth, also aligns with this [35]. However, sampling environments such as the placenta are particularly vulnerable to reagent and environmental contamination, and several large-scale analyses have questioned the existence of a placental stable resident microbiome [35]. In addition to the placenta, maternal oral and gut microbiomes have been proposed to potentially reach the fetus via the bloodstream or lymphatics, whereas vaginal microbes may ascend to the fetus through the birth canal [36, 37].

#### Trimester-specific vertical microbial transmission in GDM

Microbial changes during maternal pregnancy are influenced by hormonal fluctuations, immune modulation, and metabolic alterations. The composition and quantity of the gut microbiome differ in each trimester of pregnancy. The microbial diversity in the gut at the start of pregnancy appears similar to that of non-pregnant women, characterized by a high abundance of Bacteroidetes and Firmicutes [11, 19]. The beta diversity of the gut microbiome increases significantly during pregnancy, whereas its alpha diversity significantly declines throughout pregnancy. The abundance of butyrate-producing bacteria, such as *Faecalibacterium*, significantly decreases, whereas that of Proteobacteria and certain lactic acid-producing bacteria, including *Bifidobacteria*, substantially increases [38]. In the third trimester, the most prevalent strains observed in the gut microbiome were from the Enterobacteriaceae family and *Streptococcus* genus [11]. Some studies reported an increase in the abundance of Actinomycetota within the gut microbiome detected in meconium, as well as decreased counts of bacteria belonging to the phylum Firmicutes, specifically those in *Lactobacillus* [39, 40]. Another study observed an increased abundance of Bacteroidetes [37] and Proteobacteria [39, 41]. However, there is no literature on weekly changes in the microbiome of preterm infants born to mothers with GDM. Further research is necessary to understand how maternal GDM influences the microbial change of preterm infants.

### Mother-to-neonate microbiome transmission in GDM

Early-life gut colonization begins immediately after birth, with facultative anaerobes such as Enterobacteriaceae, *Streptococcus*, *Staphylococcus*, and *Enterococcus* dominating the first few days of life (Fig. 1; Table 1) [42]. Given these characteristics, early postnatal monitoring of the gut microbiome is crucial for understanding early-life microbial establishment. Moreover, routine fecal sampling from diapers is non-invasive and ethically sound [43]. In vaginally delivered healthy infants, Proteobacteria is initially predominant, followed by a transition to Actinomycetota [44]. The microbiome then progresses through three developmental stages, during which increasing oxygen consumption by facultative anaerobes creates an anaerobic environment favoring the growth of obligate anaerobes, such as *Bifidobacterium*, *Bacteroides*, and *Clostridium* [42, 45, 46].

Multiple factors influence the neonatal gut microbiome, including maternal GDM, obesity, diet, probiotic use, and breastfeeding [47]. Studies that control for these confounders have demonstrated that infants born to mothers with GDM exhibit lower alpha diversity, higher beta diversity, and a pro-inflammatory microbial profile [11, 37, 40, 41, 48].

At the phylum level, neonates born to mothers with GDM commonly exhibit an increased abundance of Actinomycetota [39]. However, findings on Bacteroidetes and Proteobacteria are inconsistent, with some studies reporting increased levels immediately after birth [37, 40, 49] and others describing decreases [41] in Proteobacteria [40, 50, 51]. A reduction in the abundance of Firmicutes has also been observed in GDM-associated neonates [40, 51]. Acidobacteria, Chloroflexi, and Planctomycetes were present only in controls [39]. Additionally, the abundance of Fusobacteria was significantly reduced in the neonates of mothers with GDM, even after adjusting for maternal overweight/obesity (Table 1) [51].

At the genus level, *Bifidobacterium*, *Streptococcus*, *Escherichia*, *Staphylococcus*, and members of the *Enterococcaceae* family displayed a higher relative abundance [37, 48]. Conversely, multiple studies [40, 51] found a decrease in the relative abundance of *Lactobacillus*. Similarly, the relative abundance of *Prevotella* was lower in some research Table 1) [39].

At the family level, the presence of GDM alone was associated with several differences in abundance [51]. *Lachnospiraceae* were enriched in the gut microbiome of neonates born to mothers with GDM compared with the findings for those born to non-diabetic mothers [24, 51, 52]. The relative abundance of *Lactobacillus*, *Flavonifractor*, *Erysipelotrichaceae*, and certain unspecified families in Gammaproteobacteria was decreased in the gut microbiome of children born to mothers with GDM [51].

Several studies have reported associations between maternal fasting blood glucose levels and specific microbiota. The phylum Bacteroidetes and the genus *Prevotella* were negatively correlated with maternal fasting glucose levels [39], whereas the abundance of Bacteroidetes also varied according to the maternal diabetes status [37].

### Early-life immune programming in the offspring of mothers with GDM

Human clinical and translational studies indicate that gestational diabetes mellitus (GDM) is associated with coordinated alterations in maternal metabolism and gut microbial composition that correlate with changes in early neonatal microbial trajectories and immune-related signatures; however, these findings remain largely associative, and direct causal pathways in humans have not yet been firmly established [6, 8, 31].

Recent cohort and multi-omics studies suggest that infants born to mothers with GDM may exhibit reduced microbial diversity and altered abundance of taxa such as Bifidobacterium and Faecalibacterium, alongside modest differences in inflammatory biomarkers; nevertheless, these observations are heterogeneous across populations and remain influenced by delivery mode, feeding practices, antibiotic exposure, and shared postnatal environments rather than representing uniform immune programming effects [28, 53].

Evidence regarding alterations in colostrum human milk oligosaccharides (HMOs) in mothers with GDM derives primarily from observational human studies, which report variable differences in oligosaccharide composition and potential associations with microbial colonization patterns; experimental and translational studies further suggest that specific HMOs may influence immune-regulatory microbial taxa and regulatory T-cell pathways, although definitive clinical validation of immune outcomes in neonates remains limited [54].

In contrast, mechanistic pathways linking microbial metabolites to early immune development are supported predominantly by animal and experimental studies, which propose that maternal microbiota-derived metabolites such as short-chain fatty acids may influence mucosal maturation, innate lymphoid cell development, and antimicrobial peptide expression; however, translation of these findings to human neonates remains uncertain, and terminology implying clinically validated outcomes—such as “premature immune activation”—should be interpreted as hypothesis-generating rather than evidence-based conclusions [55].

Taken together, current evidence supports a potential link between maternal dysbiosis in GDM and early-life immune-related microbial patterns in offspring, yet the persistence, clinical relevance, and mechanistic directionality of these associations remain unresolved. Future studies should prioritize longitudinal mother–infant cohorts integrating microbiome, metabolome, and immune profiling with careful control of metabolic and environmental confounders before therapeutic implications can be inferred [33, 56, 57].

## Long-term health consequences of early-life microbiome alterations in offspring of mothers with GDM

Early-life microbial composition has been associated with gastrointestinal, metabolic, immune, and cardiovascular outcomes later in life; however, these associations should be interpreted cautiously because multiple confounding factors—including maternal obesity, genetic susceptibility, dietary patterns, and underlying liver disease—may independently influence both microbiome profiles and disease risk [6, 16, 26, 53]. In offspring of mothers with GDM, observational studies suggest higher risks of metabolic syndrome, obesity, and cardiometabolic alterations; nevertheless, current human data do not establish that early microbial dysbiosis alone drives these outcomes, as maternal hyperglycemia, lipid metabolism, and intrauterine exposures likely contribute simultaneously [7].

Emerging hypotheses propose that microbiome-related metabolites may influence long-term health through interconnected gut–liver–brain signaling pathways, in which microbial products modulate hepatic metabolism, systemic inflammation, and neurodevelopmental trajectories; however, most supporting evidence remains indirect or derived from preclinical models [26, 33, 58]. Although neurodevelopmental impairments—including cognitive, behavioral, and motor outcomes—have been reported more frequently in offspring exposed to maternal diabetes, the mechanistic contribution of the microbiome remains speculative, and current studies primarily demonstrate associations rather than causation [6, 7, 59]. Similarly, immune dysregulation and allergen sensitization reported in some cohorts may reflect complex interactions among maternal metabolic status, early environmental exposures, and microbial colonization rather than alterations in the microbiome alone [28, 51, 53]. Large population-based studies have reported increased gastrointestinal morbidity among offspring of mothers with GDM; however, the absence of detailed microbiome characterization and longitudinal mechanistic data limits interpretation regarding persistence or causality [5, 60, 61].

Taken together, current evidence suggests that early-life microbiome alterations may contribute to long-term health trajectories, but these relationships remain influenced by substantial confounding variables and require carefully designed longitudinal multi-omics studies integrating metabolic, hepatic, and neurodevelopmental pathways to clarify causality.

## Microbiome-targeted therapeutic directions for GDM

**Fig. 2:**
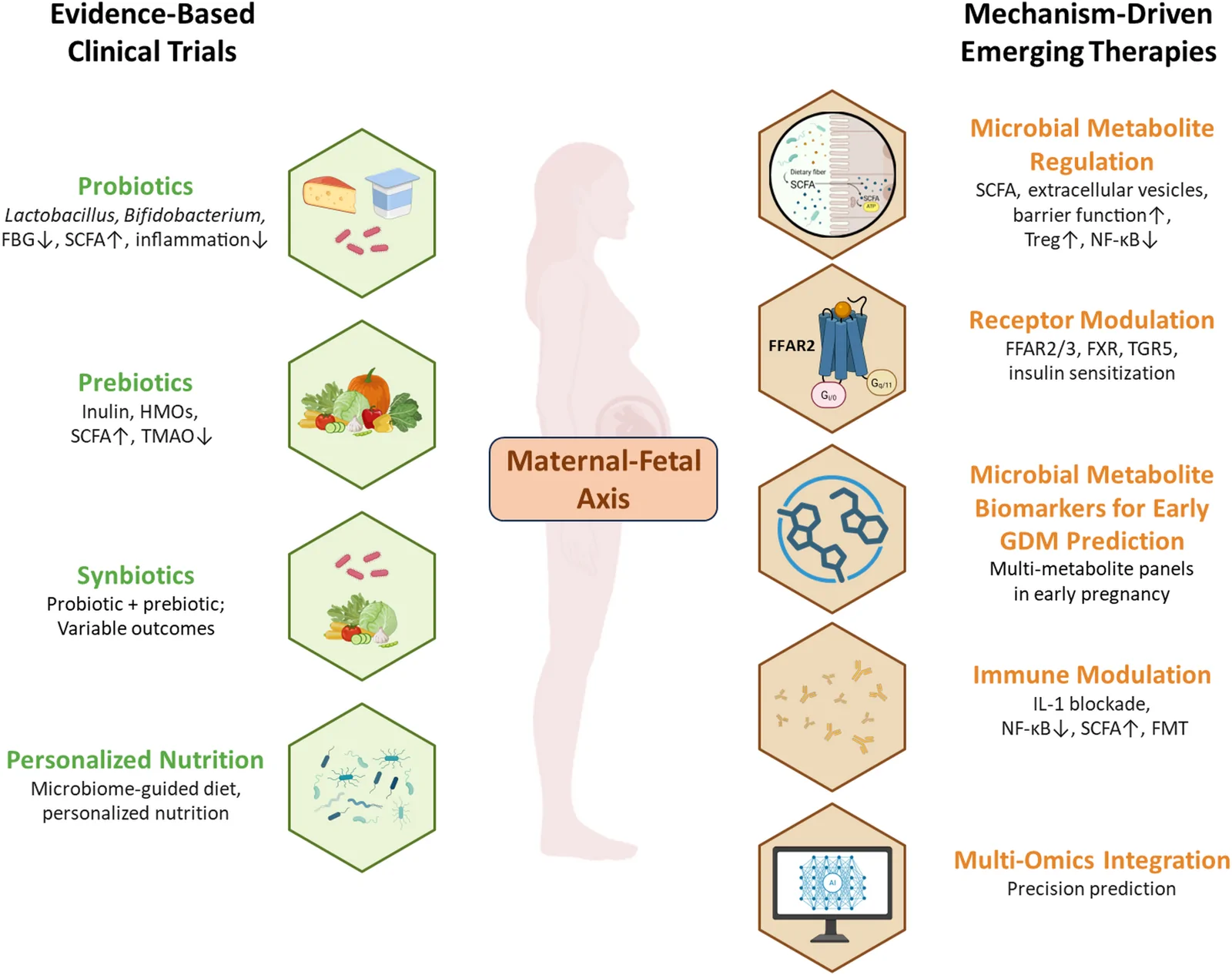
Clinical trials and emerging therapeutic strategies in gestational diabetes mellitus pregnancy. Clinical interventions include probiotics (*Lactobacillus* and *Bifidobacterium*), prebiotics (inulin and HMOs), synbiotics (probiotics and prebiotics), and personalized therapy. Probiotics can promote SCFA production while lowering fasting blood glucose level and inflammation, and prebiotics also increase SCFA synthesis, lowering oxidative stress (TMAO). The gut microbial library can provide personalized therapies, including microbiome-guided diet and personalized nutrition. Emerging therapies extend these strategies by leveraging microbial metabolites like SCFAs and extracellular vesicles to improve gut barrier function with improvement of immune responses (interleukin-1 blockade, SCFA-mediated NF-κB inhibition, and FMT) and insulin sensitization through receptor-targeted strategies, such as FFAR2/3, FXR, and TGR5. Metabolite-based biomarkers can facilitate disease monitoring in early pregnancy and precision prediction with treatment design by integrating multi-omics approaches

Recent advances in gut microbiota research have opened new avenues for the clinical management of GDM (Fig. 2). However, microbiome-targeted strategies span a spectrum of evidence levels. These include preclinical and mechanistic studies (e.g., SCFA signaling and receptor modulation) (1), early-phase clinical studies and small randomized controlled trials (e.g., probiotics and synbiotics) (2), and large multi-center precision cohorts and ongoing trials (3). While mechanistic findings provide biological plausibility, many receptor-mediated and metabolite-based strategies remain experimental and are not yet ready for routine clinical application in pregnancy. Therefore, translational interpretations in this section are categorized according to the maturity of the supporting evidence to distinguish between immediate clinical possibilities and future therapeutic targets.

### Clinical interventions—probiotic, prebiotic, and synbiotic interventions

Gut microbiota-directed interventions have been extensively investigated as adjunctive strategies to restore eubiosis and improve metabolic health in GDM (Fig. 2; Table 2). Probiotics, particularly strains such as *Lactobacillus rhamnosus*, *L. acidophilus*, and *Bifidobacterium bifidum*, are associated with improvements in fasting glucose, insulin resistance (HOMA-IR), and lipid profiles [62–65]. Their proposed mechanisms include reinforcing gut barrier function, suppressing pathogenic overgrowth, enhancing SCFA production, which in turn stimulates incretin (GLP-1), and attenuating cytokine-driven inflammation [66].

**Table 2:** Clinical interventions targeting the maternal–neonatal microbiome axis in GDM^1,2^

Category	Intervention	Proposed mechanism	Key findings	Evidence level	References
Probiotics	*Lactobacillus rhamnosus*, *L. acidophilus*, *Bifidobacterium bifidum*	SCFA production; enhancement of gut barrier integrity; modulation of GLP-1 secretion; reduction of pro-inflammatory cytokines	Associated with improvements in fasting glucose and HOMA-IR; effects on obstetric outcomes remain inconsistent	Early-phase RCTs; systematic reviews; meta-analyses	[62–66]
Prebiotics	Inulin; oligosaccharides	Selective stimulation of beneficial taxa; fermentation into SCFAs	May improve metabolic parameters, produces HMO-like metabolites; TMAO↓; Bile acid signaling; safety concerns reported in high-intake or susceptible contexts	Small RCTs; mechanistic studies	[67, 70, 71]
Synbiotics	Combined probiotic + prebiotic	Synergistic microbiota modulation; enhancement of SCFA-mediated signaling	Reported improvements in insulin sensitivity, lipid profile, and antioxidant capacity; results heterogeneous	Small RCTs; systematic reviews; meta-analyses	[65, 68]
Infant-targeted intervention	PRIMD trial	Early-life microbiome modulation	Ongoing study evaluating potential long-term metabolic and neurodevelopmental trajectories; outcome data pending	Ongoing clinical trial	[69]
Personalized nutrition	Microbiome-informed dietary modeling	Integration of microbial features into glycemic prediction models	Improved prediction of postprandial glucose variability compared with caloric models alone; clustered metabolic responses; Precision nutrition based on host-microbiome	Observational modeling studies	[72, 73]

^1^Evidence levels reflect the current maturity of available data. Reported findings are primarily associative, and clinical implementation during pregnancy requires further validation

^2^GDM, gestational diabetes mellitus; RCT, randomized controlled trial; HOMA-IR, homeostatic model assessment for insulin resistance; SCFA, short-chain fatty acids; GLP-1, glucagon-like peptide-1; TMAO, trimethylamine N-oxide; HMO, human milk oligosaccharide; PRIMD trial, Probiotics in Infants of Mothers with Gestational Diabetes Mellitus trial

Prebiotics, such as inulin and oligosaccharides, selectively stimulate beneficial taxa and are fermented into immunometabolic SCFAs [66]. Notably, certain prebiotics produce HMO-like metabolites that facilitate colonization by beneficial taxa, while also modulating bile acid signaling and reducing trimethylamine-N-oxide (TMAO) —a key metabolite implicated in insulin resistance and cardiovascular risk [67].

Consequently, synbiotics, which combine these two agents, may exhibit synergistic effects; recent RCTs have reported that synbiotic supplementation can improve not only insulin sensitivity but also lipid profiles, antioxidant capacity, and blood pressure in women with GDM [68], whereas a recent meta-analysis found no significant effect on fasting plasma glucose (FPG) [65].

Despite these promising findings, the clinical efficacy of these interventions remains debated. Meta-analyses indicate that the benefits on primary obstetric outcomes, such as cesarean section rates, insulin requirements, and neonatal birth weight, remain inconclusive [65]. This inconsistency largely reflects heterogeneity in bacterial strains, dosages, and trial designs. Moreover, while most research has focused on maternal outcomes, adequately powered trials evaluating long-term effects on offspring remain limited. Emerging protocols, such as the *Probiotics in Infants of Mothers with Gestational Diabetes Mellitus (PRIMD) trial*, are currently evaluating whether early probiotic supplementation in infants born to mothers with GDM can modulate gut dysbiosis and influence subsequent metabolic and neurodevelopmental trajectories [69].

Crucially, as these interventions move toward clinical application, potential risks and uncertainties must be considered, particularly in pregnancy. While prebiotics, such as inulin, support beneficial bacteria, high intake has been associated with exacerbation of intestinal inflammation and disease in certain individuals [70, 71]. Given the limited mechanistic data specific to pregnancy, concerns regarding placental transfer and the unintended impact on neonatal immune imprinting remain unresolved. Therefore, larger, standardized RCTs are essential to establish definitive safety protocols and dose-response relationships for these microbiota-targeted therapies.

### Personalized microbiome-targeted therapy

The growing recognition of host–microbiome heterogeneity has underscored the need for personalized nutrition strategies in GDM. Studies integrating gut microbial profiles with dietary responses have shown that individuals vary widely in their postprandial glucose trajectories despite receiving identical diets. Sugino et al. demonstrated that metabolic responses to standardized diets clustered according to host–microbiome characteristics rather than caloric intake alone [72], and Popova et al. reported that incorporating gut microbiome data into machine learning models increased the explained variance of peak postprandial glucose from 34% to 42% [73]. These findings support a shift from uniform dietary recommendations toward microbiome-informed precision nutrition, in which individualized counseling and probiotic or prebiotic regimens are tailored to a patient’s specific microbial and metabolic profile. Such approaches represent a key translational bridge between mechanistic microbiome discoveries and practical, personalized management of GDM.

### Microbiome–metabolite–immune axis as emerging therapeutic targets

Beyond established strategies, experimental approaches targeting the microbiome–metabolite–immune axis—including metabolite-based modulation, receptor-oriented strategies, immunomodulatory pathways, and multi-omics–guided biomarker discovery—are being investigated (Fig. 2; Table 3), but the supporting evidence is largely preclinical or early-phase and varies substantially across study designs [4]. Given the unique physiological context of pregnancy, therapeutic development targeting the microbiome–metabolite–immune axis requires careful consideration of maternal–fetal safety, gestational timing, and potential off-target immune or metabolic effects, highlighting the importance of a balanced risk–benefit framework [31, 74].

**Table 3:** Microbiome–metabolite–immune signaling pathways and therapeutic implications in GDM^1,2^

Target category	Key mediators	Specific receptors / pathways	Proposed mechanisms & effects	Key evidence
Metabolic signaling	SCFAs (Butyrate, Propionate)	FFAR2/3 (GPR43/41)	Triggers GLP-1/PYY secretion via Akt-STAT3 axis; improves maternal glucose tolerance; recovers GPR43 for offspring development.	[4, 7, 76]
Bile acid homeostasis	Secondary BAs (LCA, DCA)	TGR5, FXR	Activation of cAMP-PKA pathway; enhances systemic insulin sensitivity; TMAO reduction; TBA as a clinical biomarker.	[67, 74, 77]
Inflammatory axis	LPS (Endotoxemia)	TLR4-NF-κB axis	Activates RELA/CD14; establishes a self-sustaining inflammatory loop (IL-1β, IL-6, TNF-α) in the placenta; barrier disruption.	[4, 78]
Epigenetic / Tolerance	Bifidobacterium metabolites / EVs	TRIB1 methylation; Treg induction	Reduces TRIB1 DNA methylation to modulate COX-2; induces Foxp3 + Tregs; dampens Th17/Th2 programs.	[4, 7, 79]

^**1**^Pathways and mechanisms summarized in this table are derived from a combination of human observational studies, preclinical experiments, and mechanistic in vitro models. While these findings support biologically plausible links between microbiome-derived metabolites and immune–metabolic signaling in GDM, direct causality in human pregnancy has not been fully established. Therapeutic implications remain investigational and require validation in adequately powered, pregnancy-specific clinical trials

^**2**^SCFA, short-chain fatty acid; FFAR2/3, free fatty acid receptor 2/3; GLP-1, glucagon-like peptide-1; PYY, peptide YY; LCA, lithocholic acid; DCA, deoxycholic acid; TGR5, Takeda G protein–coupled receptor 5; FXR, farnesoid X receptor; LPS, lipopolysaccharide; TLR4, Toll-like receptor 4; NF-κB, nuclear factor kappa B; RELA, v-rel avian reticuloendotheliosis viral oncogene homolog A; COX-2, cyclooxygenase-2; Treg, regulatory T cell; Th17, T helper 17 cell

Accordingly, these modalities should be framed as research directions that require standardized clinical endpoints, rigorous safety monitoring, and adequately powered randomized trials—particularly when interventions could influence fetal exposure or long-term offspring outcomes [64].

#### Metabolic signaling and receptor-mediated homeostasis

Microbial metabolites, particularly SCFAs and secondary bile acids, function as key modulators of maternal-fetal metabolic homeostasis. SCFAs, generated through microbial fermentation of dietary fibers, contribute to intestinal barrier integrity by upregulating tight junction proteins such as ZO-1 and occludin and modulating immune responses [4, 75]. These SCFAs serve as essential ligands for G-protein coupled receptors, such as FFAR2 (free fatty acid receptor 2, GPR43) and FFAR3 (GPR41). In preclinical models, activation of these receptors in enteroendocrine L-cells triggers the secretion of glucagon-like peptide-1 (GLP-1) and peptide YY (PYY) through pathways such as the Akt-STAT3 axis, improving glucose tolerance by promoting insulin secretion [7].

Beyond maternal metabolism, evidence from GDM mouse models indicates that reduced maternal SCFAs diminish GPR43 signaling, which adversely affects offspring organ development, particularly kidney formation. SCFA supplementation or fecal microbiota transplantation (FMT) can improve maternal metabolic parameters and normalize offspring developmental phenotypes by recovering disrupted GPR43 signaling. While FMT represents a promising microbiome-targeted intervention, its clinical application in GDM remains highly limited and requires extreme caution. Potential safety concerns include the risk of transmitting unintended infectious agents or inducing acute immunological shifts that could adversely affect the maternal-fetal interface. Therefore, rigorous donor selection is essential, involving multi-stage screening protocols—including detailed medical history, infectious disease testing, and microbiome profiling—to ensure a ‘lean, metabolically healthy’ microbial profile without undesirable traits. Given the absence of pregnancy-specific clinical trials, FMT should be regarded as an investigational approach requiring further validation [76].

In parallel, microbial transformation of primary bile acids into secondary bile acids, such as deoxycholic acid (DCA) and lithocholic acid (LCA), serves as ligands for the hepatic farnesoid X receptor (FXR) and the intestinal Takeda G protein-coupled receptor 5 (TGR5). Binding of LCA/DCA to TGR5 on L-cells activates the adenylyl cyclase-cAMP-PKA pathway, further enhancing GLP-1 release and systemic insulin sensitivity [74]. The clinical relevance of these pathways is supported by a recent meta-analysis demonstrating that elevated serum total bile acid (TBA) levels are significantly associated with an increased risk of GDM, suggesting TBA as a potential clinical biomarker [77].

Consequently, targeting SCFA receptors (FFAR2/3) and bile acid receptors (FXR/TGR5) represents a biologically plausible therapeutic target for modulating insulin sensitivity [7, 74]. However, given the vulnerability of the maternal–fetal interface, their direct clinical application must be evaluated through a rigorous risk–benefit framework that considers gestational timing, maternal metabolic status, and potential long-term effects on offspring development, rather than relying solely on mechanistic plausibility. Therefore, current evidence supports a cautious interpretation and prioritization of well-designed prospective clinical trials to establish safety, dosing, and efficacy in GDM populations.

#### Immunomodulation and epigenetic programming

In addition to metabolic regulation, microbiome-derived metabolites exert critical immunomodulatory effects. Chronic inflammation in GDM is primarily driven by the IL-1β–TLR4–NF-κB axis within the placenta. Overexpression of IL-1 receptors (IL1R1, IL1RAP) and signaling mediators (RELA, CD14) establishes a self-sustaining inflammatory loop [78]. This pathological state is further exacerbated by maternal dysbiosis-derived LPS, which activates TLR4 signaling and promotes systemic release of pro-inflammatory cytokines such as IL-6 and TNF-α. This inflammatory profile provides multiple targets for intervention; for instance, evidence from translational studies suggests that IL-1 receptor antagonists (e.g., anakinra), anti-IL-1β antibodies (e.g., canakinumab, gevokizumab), and NF-κB/MAPK inhibitors can attenuate this inflammatory signaling and potentially hold therapeutic value in GDM.

Furthermore, beneficial taxa such as Bifidobacterium (phylum Actinobacteria) play a pivotal role in countering these inflammatory cascades through multiple immunomodulatory mechanisms. First, this genus facilitates epigenetic regulation by reducing DNA methylation of the TRIB1 gene, which subsequently modulates COX-2 expression [7]. In addition, Bifidobacterium suppresses metabolic endotoxemia by enhancing intestinal barrier integrity and increasing the bioavailability of SCFAs, particularly acetate and butyrate, which not only activate GPR41/43 to inhibit the NLRP3 inflammasome and NF-κB signaling but also modulate epithelial and antigen-presenting cell function to promote regulatory T cell differentiation and immune tolerance [4, 7, 16, 79]. Furthermore, certain Bifidobacterium-derived metabolites, including tryptophan-derived compounds, may modulate immune responses via aryl hydrocarbon receptor (AhR) signaling [4, 7, 16].

Beyond metabolite production, *Bifidobacterium* directly interacts with the host immune system to promote immune tolerance. As highlighted by Gavzy et al., these bacteria modulate dendritic cells and macrophages through cell-surface components and extracellular vesicles, leading to the induction of Foxp3 + regulatory T cells (Tregs) and attenuation of Th17 and Th2 inflammatory responses, thereby contributing to maternal–fetal immune homeostasis in the context of GDM [4, 79].

Collectively, these findings suggest that cytokine blockade and metabolite-driven immunomodulation may represent promising therapeutic avenues. However, given the immunological sensitivity of pregnancy, pregnancy-specific clinical validation and long-term safety data remain limited, requiring a cautious approach to clinical translation.

#### Multi-omics integration and metabolite-based biomarkers for precision stratification in GDM

Metabolomic profiling has consistently revealed that alterations in amino acid, lipid, and bile acid pathways emerge early, often preceding hyperglycemia (Table 4). One prospective study identified distinct metabolite profiles in early pregnancy and developed a 17-metabolite panel predicting GDM with high accuracy [area under the curve (AUC) of up to 0·97], outperforming clinical risk factors [80]. Jung et al. reported that early changes in amino acids, bile acids, and phospholipids were associated with later GDM development, with metabolic-associated steatotic liver disease (MASLD) mediating approximately 25%–32% of the effects [81]. Susarla et al. further observed that carbocyclic and branched-chain amino acids in early pregnancy and unsaturated fatty acids in mid-pregnancy were predictive of GDM, with multi-metabolite panels achieving AUCs of up to 0.98 [82]. Targeted metabolite studies indicate that higher choline levels increase the risk of GDM, whereas betaine and carnitine are protective [83]. Overall, amino acids, lipid derivatives, and microbiome-related metabolites display promise as early biomarkers and therapeutic targets; however, validation is required before their clinical application.

**Table 4:** Clinical and multi-omics studies evaluating microbiome-, metabolite-, and immune-related biomarkers in GDM^1,2^

Citation No.	Source of Population	Study Design	Sample Size & Grouping	Key Findings
[82]	PETALS cohort (Kaiser Permanente, USA; multi-racial/ethnic) + GLOW RCT validation	Prospective discovery–validation metabolomics study	Discovery: 91 GDM vs. 180 controls. Validation 1: 42 GDM vs. 372 controls. Validation 2: 35 GDM vs. 70 controls. Fasting serum metabolomics at 10–13 & 16–19 weeks	Microbiome-linked metabolites (carbocyclic acids, BCAAs, unsaturated fatty acids) strongly predicted GDM (AUC up to 0.98). Early dysbiosis-associated pathways are altered before clinical diagnosis.
[81]	Fatty Liver in Pregnancy registry, Korea	Prospective cohort with matched case–control metabolomic analysis	118 GDM cases & 118 matched controls	Early alterations in amino acids, bile acids, and phospholipids predicted future GDM. MASLD mediated 9.7–31.9% of metabolic effects.
[83]	Shanghai Birth Cohort, China	Nested case–control study	321 GDM cases & 1,284 controls (1:4, total *n* = 1605)	Higher choline increased GDM risk; higher betaine/carnitine reduced risk. Metabolite signatures correlated with OGTT glucose profiles.
[6]	Clalit HMO cohort, Israel	Prospective multi-omics cohort with mechanistic validation	Multi-omics cohort (*N* = 394) (T1 to Diagnosis)	Microbiota + SCFA + cytokine integration predicted GDM with AUC of 0.83.
[5]	GDM Mother and Child Study, China	Maternal–neonatal paired multi-omics cohort	147 GDM vs. 271 Normal (total 418 pairs)	Maternal serum metabolome alterations paralleled neonatal meconium microbiome/metabolome changes.
[87]	WeBirth cohort, Hangzhou, China	Large prospective study	1302 women with GDM	CGM-derived metrics (TAR, AUC, MBG) associated with LGA and NICU admission

^**1**^Study designs were classified according to the primary recruitment strategy and analytical framework of each study. Reported predictive performance metrics are largely derived from internal discovery and validation cohorts and require independent external validation in ethnically and geographically diverse populations before clinical implementation

^2^GDM, gestational diabetes mellitus; MASLD, metabolic dysfunction–associated steatotic liver disease; SCFA, short-chain fatty acid; BCAA, branched-chain amino acid; OGTT, oral glucose tolerance test; CGM, continuous glucose monitoring; TIR, time in range; GV, glycemic variability; LGA, large-for-gestational-age; NICU, neonatal intensive care unit; AUC, area under the receiver operating characteristic curve

Beyond single-omics analyses, multi-omics studies integrating microbiome, metabolome, and host genomics are advancing precision prediction of GDM. Longitudinal profiling has revealed that women who later develop GDM exhibit significantly reduced gut microbiota dynamics and stability as early as the first trimester, long before clinical diagnosis [21]. Combining microbiota profiles with fecal SCFAs and inflammatory cytokines has achieved high predictive accuracy (AUC = 0.83) in exploratory cohorts [6]. Furthermore, host genetic factors, such as *ADIPOQ* polymorphisms, have been linked to altered adiponectin pathways and umbilical artery flow, illustrating the contribution of host-microbiome interactions to GDM risk stratification [84].

This integrative framework extends to the maternal-neonatal interface. Coordinated alterations between maternal serum metabolites and the neonatal meconium metabolomic profiles—including shifts in taurine and bile acid biosynthesis—indicate that fetal metabolic signatures may reflect maternal dysregulation [5]. These findings support the concept of a potential “metabolic transmission axis,” although causal pathways remain to be established.

Moving toward clinical translation, interventional multi-omics studies provide insights into the complex association between diet and microbial ecology. In a randomized six-month trial in pre-diabetic adults, Shoer et al. [85] observed that a personalized postprandial glucose–targeting diet produced significantly greater shifts in gut microbiome richness, microbial pathways, serum metabolites, and cytokines compared to a Mediterranean diet. Changes in microbial composition explained 12.25% of the variance in metabolite responses, suggesting that specific microbial pathways mediate the transmission of dietary effects to glycemic outcomes. While these results offer a valuable foundation for developing precision nutrition, further validation in larger GDM-specific cohorts is required.

Currently, the Westlake Precision Birth Cohort (WPBC) has been established as an ongoing prospective initiative integrating continuous glucose monitoring (CGM), wearable tracking, and multi-omics profiling [86]. Initial analyses from this cohort demonstrated that CGM-derived glycemic metrics, such as time-in-range and glucose variability, are significantly associated with adverse pregnancy outcomes including large-for-gestational-age and neonatal intensive care unit admission [87]. The WPBC’s broader objective is to integrate these metrics with genomics and microbiome data to develop precision nutrition strategies tailored to individual metabolic responses. Taken together, metabolite-based biomarkers and multi-omics integration provide a systems-level framework for understanding GDM pathophysiology. Rather than functioning solely as predictive tools, these integrative approaches currently serve as research platforms that enhance early risk prediction and biological insight. Rigorous prospective validation and pregnancy-specific safety evaluation are required before routine incorporation into clinical obstetric practice.

## Concluding remarks and future perspectives

GDM is increasingly recognized as a multifactorial metabolic condition associated with alterations in maternal and neonatal microbiome profiles; however, current evidence primarily supports associations rather than confirmed causal mechanisms affecting immune or metabolic programming in offspring [1, 4].

From an immediate clinical perspective, management of GDM should continue to prioritize established metabolic and obstetric care strategies, while microbiome findings may currently serve as supportive mechanistic insights rather than direct therapeutic targets. Although microbiome-targeted interventions—including metabolite modulation, receptor-based strategies, and personalized probiotics—have shown biological plausibility, their clinical implementation during pregnancy remains premature due to limited randomized controlled trial evidence and unresolved safety considerations [66].

Looking forward, future research should focus on large-scale longitudinal cohorts integrating multi-omics approaches with host genetics, liver metabolism, and immune profiling to clarify causal pathways and disentangle microbiome effects from confounding maternal factors such as obesity and metabolic disease [6]. Well-designed randomized controlled trials with standardized microbial interventions, careful safety monitoring, and long-term follow-up of offspring will be essential before translating microbiome-based strategies into routine obstetric practice [64].

In summary, microbiome research offers valuable mechanistic insights into maternal–offspring health in GDM, but its primary role at present is to guide future translational research rather than immediate clinical implementation.

## Data Availability

Data sharing is not applicable to this article as no datasets were generated or analysed during the current study.
